# Examining the Levels of Violence in Mental Health Trusts

**DOI:** 10.1192/bjo.2022.188

**Published:** 2022-06-20

**Authors:** Charlotte Dinkel, Khalil Hassanally

**Affiliations:** 1 West London NHS Trust, London, United Kingdom; 2 CNWL, London, United Kingdom

## Abstract

**Aims:**

A recent NICE report stated that there were 68,683 assaults reported by NHS staff between 2013 and 2014. 69% of these were in the mental health or learning disability setting. We sought to explore the number of violent incidents within mental health trusts across England and to understand whether the levels of violence against staff have increased, decreased, or remained the same between the years 2014 to 2019. We also looked at whether a change in bed numbers correlated with the levels of violence experienced.

**Methods:**

Mental Health Trusts in England were identified, and Freedom of Information requests were sent to them. We asked for the numbers of sexual and physical violence between the years 2014 and 2020, broken down by outpatient and inpatient setting. Using bed data from NHS England we looked at whether there was a correlation with violence.

**Results:**

Out of the 53 trusts we approached with freedom of information requests, 43 returned responses with data that could be used for analysis. Data sets were often incomplete, especially for the earlier years requested. The total number of violent incidents from the 43 trusts was 24,393, in the year 2014. There was an increase to 37,907 by the year 2019, which may, in part, be explained by more complete data. Over the same time period, there was a decrease in bed numbers. Average number of episodes of violence per bed increased over 2014 to 2019 from 2 to 2.5, but the increase was not statistically significant. From our data, a correlation between the decrease in bed numbers and increase in rates of violence cannot be drawn.

**Conclusion:**

The high number of violent incidents within the mental health setting remain troubling, particularly when taking into account that this analysis represented only a partial data set. This limitation, together other data robustness issues, including the probability of under reporting by staff mean that firm conclusions cannot be drawn. This remains an area where urgent further research is needed, both to identify the extent of the problem, and to probe the impact violence has on staff and patients.

